# Undeclared work in agriculture: characteristics, estimation methods, and underlying causes

**DOI:** 10.3389/fsoc.2025.1597845

**Published:** 2025-06-10

**Authors:** Giorgia Giordani, Francesca Giarè, Simone Severini

**Affiliations:** ^1^Department of Economics, Engineering, Society and Business, University of Tuscia, Viterbo, Italy; ^2^Council for Agricultural Research and Agricultural Economy Analysis | CREA, Rome, Italy; ^3^Department of Agricultural and Forestry Sciences, University of Tuscia, Viterbo, Italy

**Keywords:** undeclared work in agriculture, agricultural employment, evaluation methodologies of undeclared work, agricultural workers, labor standards

## Abstract

Undeclared work is widespread in the agricultural labor market and very often linked to the exploitation of agricultural workers. Trying to estimate the phenomenon is rather difficult, given its hidden nature. Scholars have developed different methodologies to do this, using direct and indirect approaches. This paper aims to identify the peculiarities of undeclared work in the specific context of agriculture and the methodologies used by previous studies to assess its extent. Finally, the existing literature is analyzed to identify the reasons behind the spread of the phenomenon taking Italy as a case study. It has been observed that agriculture is mainly characterized by non-standard employment, illegal intermediation and exploitation, poor working and housing conditions, outsourced immigrant workforce, exploitation of immigrant workers, non-visibility and marginality of rural areas and a high incidence of work-related deaths. Furthermore, specific reasons seem to have a crucial influence for its widespread at the macro level. To define these aspects of undeclared work in agriculture, indirect methods have been preferred so far. Indeed, results suggest that insufficient effort has been made to understand the reasons of this phenomenon at the individual level. In particular, the extant body of literature on UW is notably deficient in addressing behavioral motivations beyond economic ones. Thus, further studies are needed to better know the phenomenon and, hopefully, to support the development more effective and efficient policies to prevent it.

## Introduction

Undeclared work (UW) was defined as “any paid activities that are lawful as regards their nature, but are not declared to the public authorities, taking into account the differences in the regulatory systems of the Member States. Applying this definition, criminal activities would be excluded, as would work not covered by usual regulatory framework and which does not have to be declared...” (Communication from the Commission to the Council, the Parliament and the European Economic and Social Commission on 24 October 2007). This definition is universally recognized as valid and has been acquired by the Member States over time.

As widely reported in the literature (Quintano and Mazzocchi, 2020; Burgstaller et al., 2022; Arezzo et al., 2024), the informal economy (within which UW is included) is difficult to determine and measure. Basically, the difficulty lies in the fact that the object of investigation is unobservable.

Aggregated and macrolevel data are provided by public institutions.

According to the European Labour Authority (Franić et al., 2023), UW in Europe accounts for ~14.8% of Gross Value Added (GVA) in the private sector, with a notable variation across member states, ranging from 5.3% in Austria to 27.1% in Romania. More specifically, Williams (2019) highlights that data from the International Labour Organization (ILO) on the EU agricultural labor force reveals that 61.2% of workers in this sector are engaged in informal employment. This contrasts with the manufacturing sector, where only 11.5% of workers are informally employed, and the service sector, which exhibits a rate of 15.4%. Furthermore, the proportion of informal workers within the agricultural sector varies substantially across the EU, ranging from just 3.4% in Sweden to as high as 91% in Poland (Williams, 2019).

So far, the efforts of researchers have been to assess the economic considerations of the actors involved in UW. In general, the existing academic literature has focused on the macroeconomic motivations of UW and the individual *economic* rationale behind the decisions of operators. However, to the best of our knowledge, there are no studies analysing the personal attitudes and social aspects of farmers toward undeclared work.

This review seeks to answer the following research questions:

- What are the peculiarities of UW in agriculture?
- What are the methodologies used to estimate the extent of UW in agriculture?
- What are the underlying causes for UW in the specific context of Italian agriculture?

To address these inquiries, the study examined institutional documents and scholarly literature on UW, with a particular emphasis on the European context. Specifically, in relation to the third research question, the analysis concentrated on literature pertaining to Italian agriculture. Given the relevance of the phenomenon, Italy allows for an in-depth exploration of UW diffusion. Therefore, this review has had an analytical purpose.

## Characteristics of the UW in agriculture

An increasing trend of non-standard employment relations is reported in agriculture, as a result of the seasonal and spot need of workforce. In fact, due to their specific characteristics, certain agricultural productions involve workers only during certain periods of the year, generally coinciding with sowing and harvesting (Williams and Horodnic, 2018; Kalantaryan et al., 2021; Battistelli et al., 2022; Corcione, 2022; Palumbo et al., 2022; Guidi and Berti, 2023; Macrì and Orsini, 2024).

Undeclared work in agriculture mainly takes the form of unregistered contracts and the exploitation of workers (Palumbo et al., 2022).

A recent report by the European Labour Authority classifies different typologies of UW relationships. These are distinguished in:

- “– Unregistered employment: an employment relationship which is not registered with the authorities when it should be registered. […]
– Under-declared employment: when formal employers pursue the illegal practice of reducing their tax and social security payments, and therefore labor costs, by under-declaring the remuneration of employees. This occurs when employers pay their formal employees two salaries: an official declared salary and an additional undeclared (“envelope”) wage which is hidden from the authorities for tax and social security purposes. Alternatively, an employer can under-declare the number of hours an employee works, such as to evade paying the minimum wage.
– Envelope wages: often used in the context of under-declared employment, an envelope wage is a cash-in-hand wage paid by a formal employer to a formal employee in addition to their official declared salary, to reduce their tax and social security payments and therefore labor costs. It arises from an agreement between the employer and employee, and additional conditions may be attached to its payment, which are not in the formal written contract or terms of employment.
– Undeclared self-employment: paid activity conducted by the self-employed where income is not declared for the purpose of evading either tax and/or social insurance contributions owed. The self-employed may not declare either some or all their income.
– Unregistered family work: labor input by individuals who are not directly paid but do contribute to the for-profit family business” (Franić et al., 2023).

These forms of UW are recorded in agriculture.

Also, illegal intermediation between the employer and employees, known as “caporalato,” is a widespread phenomenon (Corrado et al., 2018; Omizzolo, 2019; Battistelli et al., 2022; Perrotta and Raeymaekers, 2023). Undeclared workers often face worse working conditions, lower pay, violations of their labor rights, and limited protection under labor and social laws. As a result, they miss out on essential social benefits, pension entitlements, healthcare, and opportunities for skills development and lifelong learning (Williams, 2020; Guidi and Berti, 2023; Macrì and Orsini, 2024). The picture painted is exacerbated by the fact that the average annual number of work-related deaths in agriculture is 170,000 (FAO figures, worldwide; Yeshanew, 2018).

**Figure 1:**
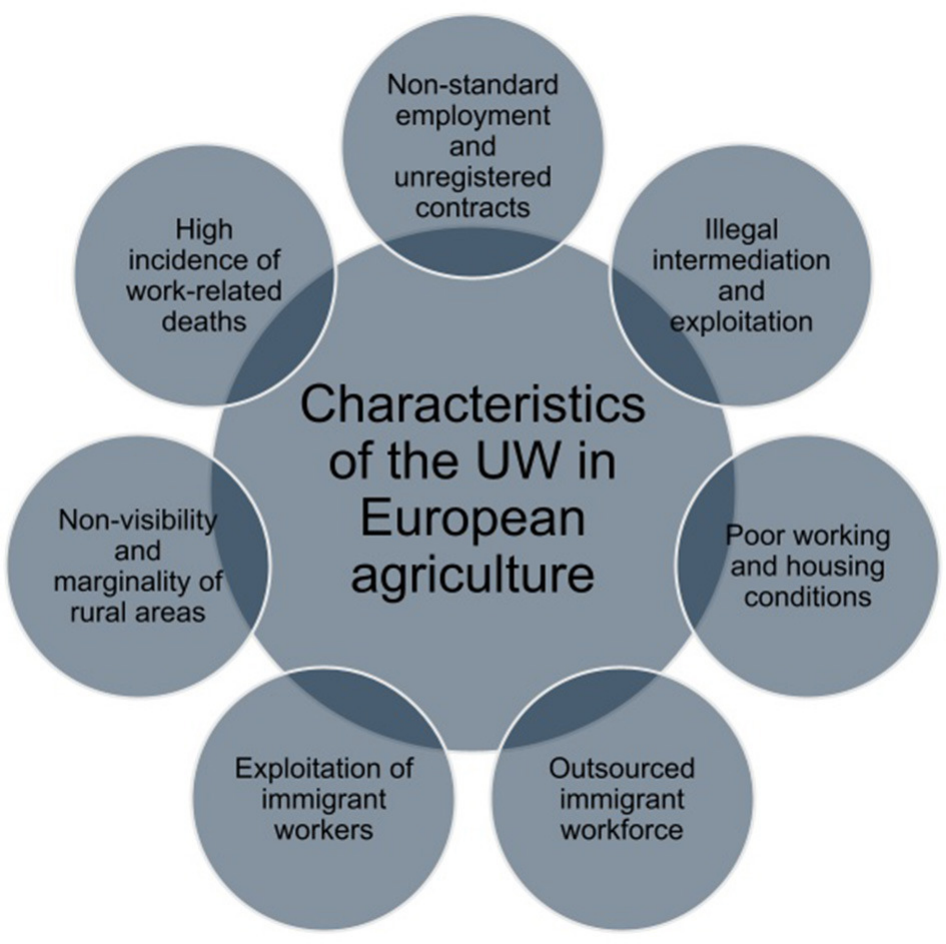
Overview of characteristics of UW in European agriculture. Source: own elaboration.

In light of contemporary global socio-economic dynamics, it is posited that the European agricultural labor force is increasingly delineated by a decline in familial employment and a concomitant rise in the utilization of outsourced labor (Williams and Horodnic, 2018; Kalantaryan et al., 2021). Furthermore, this labor demographic is predominantly comprised of migrants who traverse national borders in pursuit of employment opportunities (Corrado et al., 2018; Antonioli et al., 2023; Palumbo et al., 2022; Guidi and Berti, 2023). With regard to immigrant workers, Corrado et al. (2018) reported that “Rural areas also offer degrees of non-visibility and informality that help accommodate migrants with different types of legal status, although this simultaneously paves the way for irregular practices and situations of harsh exploitation.” The marginality of the countryside also contributes to the spread of poor housing conditions. Indeed, the drama of the *ghettoization* of migrant workers, who are forced to spend their non-working hours in huts or warehouses (sheds) with absolutely unacceptable hygienic conditions and not suitable for human sustenance, is widely known in Italy (Giovannetti et al., 2022). These inhumane conditions have in some cases aroused the anger of foreign workers, to the point of real rebellions (Figure 1; Gaudio et al., 2020).

Therefore, in general, agriculture is one of the most representative sectors where workers can suffer from poor working and living conditions. Estimating its extent is not easy, although the interest in developing policy measures to combat it has led scholars to analyze it. In the following section, we summarize the main methodologies used so far.

## Estimating the UW in agriculture

**Figure 2:**
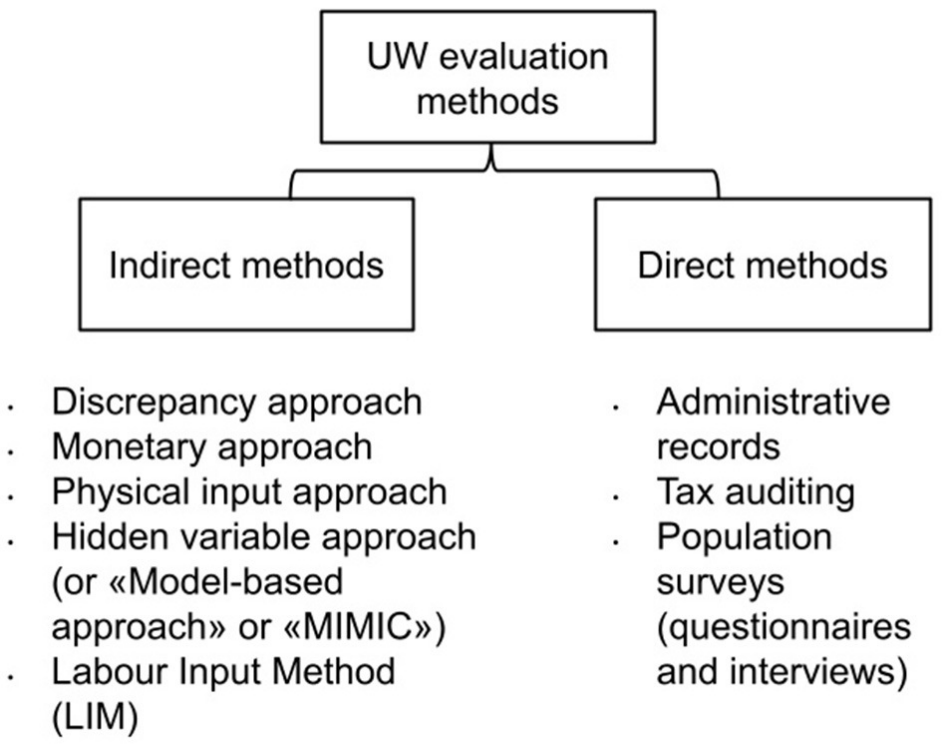
UW evaluation methodologies. Source: own elaboration.

Undeclared work is usually estimated using indirect or direct methodologies, at macro or micro (individual) level (Figure 2; Kirchner, 2014; Williams, 2020; Arezzo et al., 2024). These different methods result in very heterogeneous estimates.

Indirect methods are mostly based on macroeconomic indicators. They can be summarized in:

- *Discrepancy approaches*: they consist in comparing the expenditure measure of gross national product with its income measure (assumed to be equal in the formal economy). Otherwise, the comparison can also be made between the official and the actual labor force.
- *Monetary approaches*: shadow economic activities are settled by paying cash in order not to be traced. Therefore, with these types of surveys, tracking work is usually carried out or credit card transactions are imposed to assess changes in the circulation of money.
- *Physical input approaches*: for example, electricity consumption approach belongs to the class of physical input methods. It uses electricity as indicator of economic activity. Indeed, the indicator provides information for evaluating the actual size of a firm and its actual need of workforce.
- *Hidden variable approaches*: also referred to as “model-based approaches,” these methods allow for the inclusion of multiple causes and multiple observable indicators. This approach is commonly known as the “MIMIC” (Multiple Indicators Multiple Causes) method.

Furthermore, the European Platform Tackling Undeclared Work (established by the European Commission in 2016) started using the so-called Labour Input Method (LIM), developed by the Italian Institute of Statistics “ISTAT” (Calzaroni, 2000; Williams et al., 2017; Søndergaard, 2023). The LIM estimates undeclared work by measuring the discrepancy between reported labor supply and demand.

Again, several measurement approaches are subsumed within direct methods: the use of administrative records, tax auditing, as well as the use of population surveys (Kirchner, 2014). Direct survey methods are advocated to identify its characteristics in terms of who engages in undeclared work, what they do and why, so as to inform policy development (Williams, 2020).

Among the approaches mentioned above, the experimental survey techniques (questionnaires and interviews) seem to “increase the validity of the measurement of undeclared work and allow for the causal identification of its determinants” (Burgstaller et al., 2022). Furthermore, with representative surveys it is possible to analyze moral attitudes of respondents (AitBihiOuali and Bargain, 2021; Burgstaller et al., 2022) and implement administrative data information (De Gregorio and Giordano, 2016).

Some researchers agree on the limitations of direct methods. One of the main problems is that, unlike indirect methods, they underestimate actual behavior (Arezzo et al., 2024). Indeed, as widely reported (Kirchner et al., 2012; Kirchner, 2014; Burgstaller et al., 2022; Arezzo et al., 2024), given the sensitive topic, respondents reporting about undeclared work may suffer from dishonest answering behavior, referred to as social desirability bias (SDB).

A second important aspect that poses a challenge to the direct method is the wording used to assess individual opinions. In fact, there is a large body of research on how best to design questionnaires and interviews to make them scientifically valid (Kirchner et al., 2012; Kirchner, 2014).

Moreover, cross-country comparisons and considerations may be difficult given the contextual specificity of such studies (Burgstaller et al., 2022).

Nonetheless, direct methods offer the possibility of studying undeclared work specifically, without having to consider the full range of activities in the informal economy. In addition, specific economic sectors can be analyzed (Kirchner, 2014).

One of the few recent and comprehensive studies on UW in the European agriculture is that of Williams and Horodnic (2018). It is based on data from institutional reports and the results of previous surveys, but the authors contributed by developing several statistical elaborations. Also, Schneider et al. (2023) applied the MIMIC model, using 4 influencing factors (total tax burden, share of imported agricultural goods, share of subsidies, factor income in agriculture) and 2 indicators (GVA and agricultural employment rate). The study was designed as a cross-country analysis and data for the 4 influencing factors were taken from institutional databases (aggregated data). The same was done for the two indicators.

The use of direct methods is very rare. In fact, to the best of our knowledge, no interviews or questionnaires have been used to estimate UW in agriculture in the last decade, with a few exceptions: Lord's work (Lord, 2019) aimed to investigate the interaction between legislation and informal norms in the UK agricultural labor market by interviewing farmers and agricultural employees. In particular, three legal provisions related to wages were analyzed: pensions, housing and apprentice wages. Personal attitudes and social aspects were not considered in this analysis.

Furthermore, Macrì and Orsini (2024) analyzed some Italian policy instruments created to counteract UW in agriculture. The authors provided a qualitative summary of their survey on a sample of Italian farmers. The study aimed to understand farmers' adherence and consideration regarding available legal forms of agricultural workforce recruitment.

## Underlying causes for UW: the case of Italian agriculture

Three main existing theories on the macroeconomic causes underlying undeclared work can be mentioned: modernization theory, neoliberal theory and political economy theory (Williams, 2020; Williams and Horodnic, 2020). The first refers to the absence of public incentives for welfare and the lack of administrative efforts to improve labor market governance. The second considers tax pressure and in general bureaucratic limitations to business management. The third, on the other hand, is based on the general state of inertia of public administrations and control systems toward corruption and negative behaviors of economic operators. Williams (2020) has shown that these theories are often non-exclusive and that specific causes can be identified with respect to the country considered.

The extent of the UW in Italy is widely acknowledged, especially in the agricultural sector: the “Placito Rizzotto” Observatory has reported that the share of undeclared workers in agriculture is estimated at around 16.8%, or 205,800 full-time equivalent work units (VII Report on FLAI-CGIL, 2024).

Several scholars refer that one of the main reasons is the so-called “squeeze on agriculture” (Corcione, 2022). This means that there are strong bargaining inequalities between actors in the agri-food supply chains and that farmers are underpaid for their productions in the face of buyers' demands for out-of-contract discounts (Corrado et al., 2018; Battistelli et al., 2022; Macrì and Orsini, 2024). In this scenario, farmers adopt two solutions to reduce labor costs: Williams calls them the 'high road' and the 'low road' (2019). When they have the economic availability, they can choose to implement their labor force with mechanization (high road). Otherwise, they hire their employees irregularly (low road; Battistelli et al., 2022).

Another important factor contributing to UW in Italian agriculture is the failure to use official channels for matching labor supply and demand. Indeed, although the illegal recruitment is punished by the law (Art. 603bis of the Penal Code and Law n. 199/2016) and public job centers and online platforms are provided, illegal ways of recruitment are preferred (Battistelli et al., 2022; Macrì and Orsini, 2024).

Some scholars also added that the Italian policy on the entry of migrant workers has determined and continues to determine the phenomenon of UW, since it is designed without taking into account the conditions of asylum seekers and the contemporary internal need for agricultural labor (Corrado et al., 2018; Leccese and Schiuma, 2018; Corcione, 2022; Macrì and Orsini, 2024).

For their part, migrant workers are often unaware of their “rights” and of “social bonds.” Therefore, they are most vulnerable (Corrado et al., 2018; Palumbo et al., 2022). Guidi and Berti (2023) reported that “the lack of language proficiency, the scarcity of information, the legal precariousness, the migratory debt, and the need to send remittances back home, along with widespread fear, are some of the factors that contribute to the vulnerability of migrants in the territory.”

The presence of UW in the Italian agricultural sector is also due to the institutional and legal management of this phenomenon (Leccese and Schiuma, 2018; Battista, 2022; Battistelli et al., 2022).

In particular, one of the weak points is the system of collective agreements. These, in fact, are not homogeneous throughout the national territory (there are different regional and local agreements) and this “makes it difficult for a worker to precisely know his rights, and his pay rate” (Battistelli et al., 2022).

Considering other public interventions, in 2018 the Italian government established the “Operational Table for the definition of a new strategy to combat gangmastering [caporalato] and labor exploitation in agriculture.” The Table committed to defining a strategy to combat gangmastering and this objective was defined in a Three-Year Plan to combat the phenomenon. The plan included six macro-areas of intervention, including the Quality Agricultural Work Network. This network (established by Law 116/2014) allows Italian farmers to voluntarily register their farms and represents a way to demonstrate compliance with labor standards. However, Macrì and Orsini reported that farmers' membership in the network was mainly driven by their desire to avoid inspections and by requests from their retailers. Overall, Macrì and Orsini's study reported a low level of membership in the network and some interviewees stated that they had not joined because they were unaware of it.

Finally, social macrolevel aspects can be mentioned: Williams defines “informal institutions” as those dynamics of civic life in which people behave in a certain way based on the consensus of their peers and commonly accepted norms, not institutional but dictated by a specific lifestyle of the place. Therefore, in geographical areas where corruption is historically rooted, UW is more likely: in the report by Franić et al. (2023), Italy is among the countries in which the extent of UW is negatively proportional to trust in public institutions and social capital (used as an indicator of trust among peers).

## Discussion and conclusion

The undeclared work in agriculture represents a real and serious problem. Social changes contribute to the phenomenon. It has well defined characteristics and causes and sees immigrant workers victims of harsh working treatments.

Therefore, structural political interventions are necessary. This is not valid only for the Italian context, but for the European and global agricultural sector, more generally.

For this reason, the efforts of research on undeclared work have a crucial value. According to Robert (2011), a clear undeclared work definition is important to understand his own area, create an effective legal and administrative response and guarantee coherent treatment. Our work has revealed that indirect methods are preferred by experts to estimate UW in agriculture. Further studies should be made to analyze individual motivations (micro level approach) that determine the UW in agriculture. In fact, this seems to be a gap in existing literature. In particular, although we know, the moral attitudes and social motivations of farmers have never been evaluated in terms of conformity to work standards, nor their information and awareness of the labor laws, using a direct method. As Williams (2020) pointed out, knowing who is involved in UW can be a valid tool for the implementation of strategies to face the UW.

However, based on the macroeconomic reasons underlying the phenomenon, found so far in the Italian context, it would be appropriate to address the anomalies in the labor recruitment system. This requires a strengthening of the control system and the promotion of public and private platforms for the matching of supply and demand (Leccese and Schiuma, 2018; Macrì and Orsini, 2024). Furthermore, it would be useful to promote legal bargaining processes by harmonizing current territorial collective agreements. Last but not least, it would be important to contribute to the balancing of bargaining power along the agri-food supply chains, so as to discourage the abusive use of agricultural labor.
